# Porcelain Inlays from the Standpoint of a Country Dentist

**Published:** 1906-03

**Authors:** J. L. Bridgeford

**Affiliations:** Macon, Mo.


### PORCELAIN INLAYS FROM THE STANDPOINT OF A COUNTRY DENTIST.

By J. L. Bridgeford, D. D. S., Macon, Mo.

This short paper will not go into details and discuss methods and principles of inlay work, for unless one has something new to offer he would be only threshing over old straw. My aim, therefore, shall be to answer the following questions that have been asked: Can a charge be made for the work that will be high enough? Will the people of the smaller towns want this work, and can the country dentist accomplish these operations as satisfactorily as does his city colleague?

At the outset I wish to say "Yes" to all questions.

As to the charge allow me to say, that any man is worthy of his hire, and when a dentist is called on to do a given operation, he owes it to his patient as well as to himself, to charge that patient a fee that will enable him to discharge that sacred trust which has been placed in him, and give to his patient the benefit of the best skill and knowledge at his command. I will admit that the fees of a country dentist are not so large, perhaps, as those of a city practitioner, but conditions are such that do not warrant it. The rents are not so much, one's living expenses are not so high and perhaps his clientage not so wealthy as those of his city confrere. But suffice it to say, about as many country dentists die rich as those of the city since not many of either class are over-burdened along this line. It has not been many years ago that the idea prevailed among many of the rural people, to have gold in the teeth in any form was evidence of aristocracy, but when the hod-carrier, the colored coachman, and the Pulman porter joined this class of modern gentry the cry for something less conspicuous and more artistic went up, and has continued and will continue.

There are no longer in this rural world of ours the back woodsman as in former times. Uncle Sam has made it possible for him to have the daily paper and other educational matter delivered at his door each morning the same as if he lived in the city. The telephone has placed him in speaking distance with the world. In the universities and colleges are to be found their boys and girls. The public highways are receiving more attention thereby making social intercourse more inviting and the country as a whole more attractive, especially since the country is much more thickly populated than even a few years ago.

It has been my experience that the people in the smaller towns are much more ready to accept porcelain inlays than the dentists in the same places.

Now, why is this? Porcelain certainly has been tested and while perhaps not found to be a cure-all it certainly has its place in dentistry and should be in the hands of every dentist.

The four years of my experience in porcelain work has not been all sunshine but today I can point with pride to operations that as a beginner I thought most impossible.

The country dentist is just as capable and should be the peer of any dentist if he equips himself and puts the equipments into use.

Porcelain work was born to live.

